# The developmental miR-17–92 cluster and the Sfmbt2 miRNA cluster cannot rescue the abnormal embryonic development generated using obstructive epididymal environment-producing sperm in C57BL/6 J mice

**DOI:** 10.1186/s12958-022-01025-x

**Published:** 2022-11-30

**Authors:** Xunwei Wu, Xiaomei He, Qian Liu, Honggang Li

**Affiliations:** Institute of Reproductive Health, Tongji Medical College, Huazhong University of Science and Technology, Wuhan, People’s Republic of China

**Keywords:** miRNAs, Epididymis, Embryonic development

## Abstract

**Background:**

Sperm, during epididymal transit, acquires microRNAs(miRNAs), which are crucial for embryonic development. However, whether sperm miRNAs influenced by an obstructive epididymal environment affect embryonic development remains unknown.

**Method:**

The sham operation and vasectomy were performed in C57BL/6 J mice to create the control group (CON) and the obstructive epididymal environment group(OEE) group, respectively. The morphology of the testis and epididymis was observed using hematoxylin and eosin staining (HE staining) to establish the OEE mice model. The sperm quality test, intracytoplasmic sperm injection (ICSI), and epididymosomes fusion were employed to observe the effect of the obstructive epididymal environment on sperm and resultant embryonic development. The alteration of the sperm small RNA (sRNA) profile was analyzed by sRNA sequencing. RT-qPCR and DNA methylation were applied to observe the effect of obstructive epididymis on the expression of sperm miRNAs. The miRNAs microinjection was used to explore the impacts of sperm miRNAs on embryonic development.

**Results:**

We confirmed postoperative 8-week mice as the OEE mice model by examining the morphology of the testis and epididymis. In the OEE group, we observed that sperm quality degraded and the development potential of embryos was reduced, which can be saved by the normal epididymal environment. The sperm sRNA sequencing revealed that the expression of the developmental miR-17–92 cluster and the Sfmbt2 miRNA cluster was downregulated in the OEE group. The expression of these two miRNA clusters in epididymis was also downregulated and regulated by DNA methylation. However, the downregulation of either the miR-17–92 cluster or the Sfmbt2 miRNA cluster in normal zygotes did not impair embryonic development.

**Conclusion:**

The obstructive epididymal environment influences sperm quality and resultant embryonic development, as well as the abundance of the developmental miR-17–92 cluster and the Sfmbt2 miRNA cluster in sperm, but these miRNA clusters are not the cause of abnormal embryonic development. It implies that epididymis is important in early embryonic development and may play a potential role in sperm epigenome.

**Supplementary Information:**

The online version contains supplementary material available at 10.1186/s12958-022-01025-x.

## Background

Obstructive azoospermia (OA) results in 40% of male infertility. It is characterized by the absence of sperm in the ejaculate due to reproductive tract occlusion, but it has normal testicular and endocrine functions [1]. It accounts for approximately 1% of all men and 10–15% of infertile men [2]. The clinical treatment for OA includes microsurgical reconstruction of the reproductive tract and sperm retrieval for ICSI (An infertility treatment that involves injecting sperm into oocytes to assist fertilization) [3]. The obstruction of OA patients’ reproductive tract can occur in the rete testis, efferent ducts, epididymis, vas deferens, and ejaculatory duct (listed in order of the path of sperm ejaculation). However, the most common region is vas deferens [4, 5], which means most OA patients’ post-testicular sperm maturation is in an abnormal epididymal environment. The debate over whether the obstructive epididymal environment affects sperm and results in abnormal embryonic development has continued; nevertheless, no definite conclusion has been drawn regarding this issue [6–10].

The epididymis is an essential organ for post-testicular sperm maturation, where sperm acquires proteins, sRNAs, forward motility ability, and fertilization capability [11]. Colin's study reveals that epididymis affects embryonic development, as the caput sperm can’t support the post-implantation development of the embryo due to a lack of miRNAs transmitted from epididymis [12]. The high throughput sequencing (NGS) of sperm from various developmental stages reveals that the abundance of miRNAs changed during epididymal maturation [13]. Previous researches have found that the epididymis influences the sperm miRNA profile via epididymosomes [13–16]. However, whether sperm miRNAs influenced by an obstructive epididymal environment affect embryonic development remains unknown.

MiRNAs are a subset of non-coding RNAs, 19–25 nt long in size. It participates in a series of biological processes through silencing target genes, such as differentiation, proliferation, and apoptosis [17, 18]. For decades, increasing evidence has suggested that sperm miRNAs play a pivotal role in epigenetic inheritance [19–22]. It acts as a carrier of epigenetic inheritance affecting early embryonic development. The miRNA-34c and miRNA-449b have been reported to be required for early embryo cleavage [23, 24]. DROSHA and DICER are RNase III enzymes that process primary miRNAs (pri-miRNAs) and precursor miRNAs (pre-miRNAs). Conditional knockout (cKO) of DICER or DROSHA in the testis disrupts miRNA synthesis, resulting in abnormal early embryonic development, whereas injecting normal sperm RNA into these embryos rescues their development [25]. Furthermore, miR-880 cluster, miR-17–92 cluster, miR-106b-25 cluster, and miR-34b/c delivered from the epididymis to sperm were reported to be essential to post-implantation embryonic development [12]. It is unclear what kind of sperm miRNAs may play a role in the embryonic development of obstructive epididymal environment-producing sperm.

To investigate whether an obstructive epididymal environment affects sperm miRNAs and resultant abnormal embryonic development, we developed a mice model to mimic an obstructive epididymal environment by vasectomy. The current study identified that the obstructive epididymal environment affected sperm quality and resultant embryo development, as well as sperm miRNA profile.

## Materials and methods

### Animals and establishment of the mice model

Male C57BL/6 J mice were purchased from the Experiment Animal Center, Center for Disease Control and Prevention, Hubei Province (Wuhan, China), female B6D2F1 mice (C57BL/6 J: BDA/2 F1 hybrid background) and ICR mice were purchased from Charles River Laboratories (Beijing, China). All mice were housed in the Laboratory Animal Center of HuaZhong university of science and technology, fed a chow diet ad libitum, and kept on a 12:12 light: dark cycle with a temperature of 22 °C and relative humidity of 42%. Sham operation and vasectomy were performed on 6-week-old male C57BL/6 J mice to create the CON and OEE groups. Male C57BL/6 J mice were anesthetized with 3.3% chloral hydrate, and the vas deferens were ligated by a suture line in the OEE group; the vas deferens in the CON group were not ligated. The CON group and the OEE group mice were euthanized in the postoperative period at 4, 8, and 12 weeks, and the epididymis and testis samples were collected and weighed. The study was approved by the Animal Care Ethics Committee of Huazhong university of science and technology.

### HE staining

The epididymis and testis were fixed in 10% neutral buffered formalin and embedded with paraffin. The paraffin section was stained with hematoxylin and eosin to observe the morphology.

### Sperm isolation and sperm quality test

Sperm was released from cauda epididymis in prewarmed G-IVF PLUS (Vitrolife AB, Goteborg, Sweden) at 37℃ for 30 min. After blending gently, 30 ul of sperm suspension was divided into three equal portions. One was for examining sperm concentration and motility, one was for observing sperm viability stained with eosin staining, and the last one was stained with Diff-Quick staining (Nanjing Jiancheng Bioengineering Institute, Nanjing, China) for observing morphology. The sperm DNA fragmentation index (DFI) was determined by flow cytometry (BD bioscience, San Jose, CA). Sperm were diluted to a concentration of (1–2) × 10^6^/ml with cold TNE buffer (Servicebio Technology, Wuhan, Hubei, China), stained with acridine orange solution (Sigma-Aldrich, Shanghai, China), and then tested with flow cytometry (BD Biosciences, Franklin Lakes, USA).

### Sperm preparation for ICSI

Sperm was optimized by density gradient centrifugation using a Sydney IVF sperm gradient kit (Cook Medical, Sydney, Australia). The gradients were prepared by adding 1.5 mL of 40% solution to 1.5 mL of 80% solution in a 4 ml centrifuge tube and prewarmed at 37℃. The sperm suspension was gently added to the gradient solution and centrifuged at 600 g for 15 min. The collected sperm precipitate was washed by G-IVF PLUS twice and resuspended by 500 μl G-IVF PLUS. The sperm head was separated by a 30% power output of ultrasonic sonicator for 2 min; then, sperm head suspension was stored at -80℃.

### Epididymosomes isolation and fusion

The epididymis was minced and immediately transferred to prewarmed PBS at 37℃ for 30 min after being collected from mice, and the suspension was filtered through 100 μm and 40 μm cell strainer to collect the supernatant. The filtered supernatant was performed in sequential centrifugation (centrifuge at 300 g, 2000 g, 10000 g, and 100000 g for 10 min, 15 min, 30 min, and 80 min, respectively). After centrifugation, the precipitate was reserved and resuspended with G-IVF PLUS to incubate with optimized sperm for 4.5 h. Sperm was collected by centrifuging 600 g for 15 min and prepared by sonication for injection.

### ICSI and embryo transfer

Intraperitoneal injection with 8 IU pregnant mare serum gonadotropin (PMSG) (NINGBO SANSHENG, Ningbo, China) was performed in 6–8-week-old B2D6F1 female mice at 5 pm; after 48 h, intraperitoneal injection with 8 IU human chorionic gonadotrophin (hCG) (NSNF, Ningbo, China). Oocytes were collected 12-14 h after hCG administration, and the cumulus-oocyte complexes (COC) were washed in prewarmed G-MOP PLUS medium (Vitrolife AB, Goteborg, Sweden) containing 0.4 mg/ml hyaluronidase. Mature oocytes (with the first polar body) were selected for injection. ICSI microinjection needle (WPI, TW100-4, Sarasota, USA) was pulled by Flaming Micropipette Puller System P-1000(Sutter, Novato, CA, USA). ICSI was manipulated with the Eppendorf PiezoXpert®(Eppendorf AG, Germany) following standard ICSI procedures [26, 27]. The zygotes were then cultured in G1-PLUS/G2-PLUS sequential medium (Vitrolife AB, Goteborg, Sweden) at 37 °C in 5% CO2. The female ICR mice were mated with male ICR mice at 5 pm, and the female mice with vaginal plugs were chosen as surrogates. The two-cell embryos were transferred to the ampulla oviduct of female ICR mice.

### Sperm RNA extraction

After oozing from the epididymis, sperm were successively filtered with 100 μm and 40 μm cell strainer to remove the tissue debris. Somatic cell lysis buffer (0.1% SDS, 0.5% Triton X in DEPC H_2_O) was used to wipe out somatic cell contamination and then treated with sperm lysis solution (61.1% Guanidine-HCl, 3.5%EDTA, 1.5%tris, 20%Tween-20, 5%Triton in DEPC H_2_O) to lyse sperm utterly. Sperm lysis was transferred to 800 μl TRIzol LS reagent (No. 10296028; Invitrogen; Thermo Fisher, Waltham, MA, USA) and blended vigorously. After placing in ice for 1 h, 200 μl chloroform was added and placed in ice for 10 min. Then the mixture was centrifuged at 12 000 g for 15 min at 4 °C to collect the aqueous phase. 500 μl isopropanol was added to the aqueous phase and incubated at − 20 °C for 30 min. The precipitate was collected by centrifuging at 12 000 g for 15 min at 4 °C and washed with 75% ethanol. The final products were resuspended with RNase-free water, and the RNA concentration was measured by Nanodrop 2000(Thermo, San Jose, CA, USA).

### sRNA sequencing

Sperm RNA isolated from OEE and CON groups were used to build a library of sRNAs. Agarose electrophoresis was used to test the integrity of total RNA samples. Total RNA samples were first pretreated with NEBNext® Multiplex Small RNA Library Prep Set (Illumina, USA) for library construction. Some RNA modifications that could interfere with library construction were removed as follows: Deacetylation of 3'-aminoacyl (charged) to 3'-OH for 3' adaptor ligation, removal of 3'-(2',3')-cyclic phosphate to 3'-OH for 3' adaptor ligation, phosphorylation of 5'-OH to 5'-P for 5'-adaptor ligation, and demethylation of m1A and m3C for efficient reverse transcription. The Agilent 2100 Bioanalyzer was used to quantify the completed libraries. The sequencing run was performed on the NextSeq system using NextSeq 500/550 V2 kit. Sequencing was carried out by running 50 cycles. Image analysis and base calling were performed using Solexa pipeline v1.8 (Off-Line Base Caller software, v1.8). FastQC examined sequencing quality, and trimmed reads are aligned, allowing for 1 mismatch only to the mature tRNA sequences, then reads that do not map are aligned, allowing for 1 mismatch only to precursor tRNA sequences with bowtie software. The remaining reads are aligned, allowing for 1 mismatch only to miRNA reference sequences with miRDeep2. The differentially expressed tRFs & tiRNAs and miRNAs were screened based on the count value with R package edgeR and performed in R. All steps in the library construction, sequencing, and bioinformatics analysis of sRNAs were performed by KangChen Bio-tech (Shanghai, China).

### Real-time quantitative polymerase chain reaction (qRT-PCR)

QPCR of sperm miRNAs were using the All-in-one miRNA qRT-PCR Detection Kit (GeneCopoeia, Maryland, USA). Reverse transcription and qPCR of genes were respectively using HiScript II Q RT SuperMix for qPCR (Vazyme, Nanjing, China) and ChamQ Universal SYBR qPCR Master Mix (Vazyme, Nanjing, China). cDNAs obtained were diluted for qPCR. All primers of miRNAs and genes are listed in Supplementary Table 1. Amplifications were performed at least triplicate for each sample. Relative gene expression levels were evaluated using the 2^−ΔΔCt^ method and normalized to U6 Small nuclear RNA (snRNA) level or beta-actin (Actb).

### DNA methylation

Epididymis DNA was extracted using TIANamp Genomic DNA Kit (TIANGEN, Beijing, China). Genomic DNA (500 ng) was bisulfite-treated using the EZ-DNA methylation kit (Zymo, Irvine, CA). The interested DNA sequence was amplified using SYBR Premix TaqTM qPCR Reagent Kit (TAKARA, Beijing, China) with the bisulfite-treated DNA as the template. Interesting sequences were located in the promoter region of *Sfmbt2* and *MIR17HG*. Primer information was included in Supplementary Table 2. Agarose electrophoresis was performed to purify and collect amplified cDNA. The methylation level was detected by absolute quantification qPCR using purified cDNA as a standard sample.

### Synthetic miRNAs microinjection

Synthetic miRNA inhibitors and scrambled RNA were manufactured by Sangon Biotech (Shanghai, China), listed in Supplementary Table 3. Equal amounts of miRNA inhibitors were mixed and adjusted to a concentration of 2 ng/μl. The microinjection needle was pulled using borosilicate glass (Sutter, Novato, CA, USA) in Flaming Micropipette Puller System P-1000. Superovulation was conducted in the female B2D6F1 mice. The female B2D6F1 mice were mated with male C57BL/6 J mice to collect zygotes for miRNAs microinjection. MiRNAs microinjection was performed in a G-MOP PLUS medium. Synthetic miRNA inhibitors and scramble RNA as control were microinjected into the zygotes with two pronuclei by Femtojet 4i (Eppendorf AG, Germany) [28].

### Statistical analysis

All statistical analyses were performed using Graphpad Prism 8.0 (La Jolla, CA, USA). The experimental results are presented as mean ± standard error of the mean (SEM). Data were analyzed using the t-test, Mann–Whitney analysis, or chi-square test. *P* < 0.05 was considered statistically significant.

## Results

### The alteration in the testis and epididymis of different obstructive interval

**Fig. 1:**
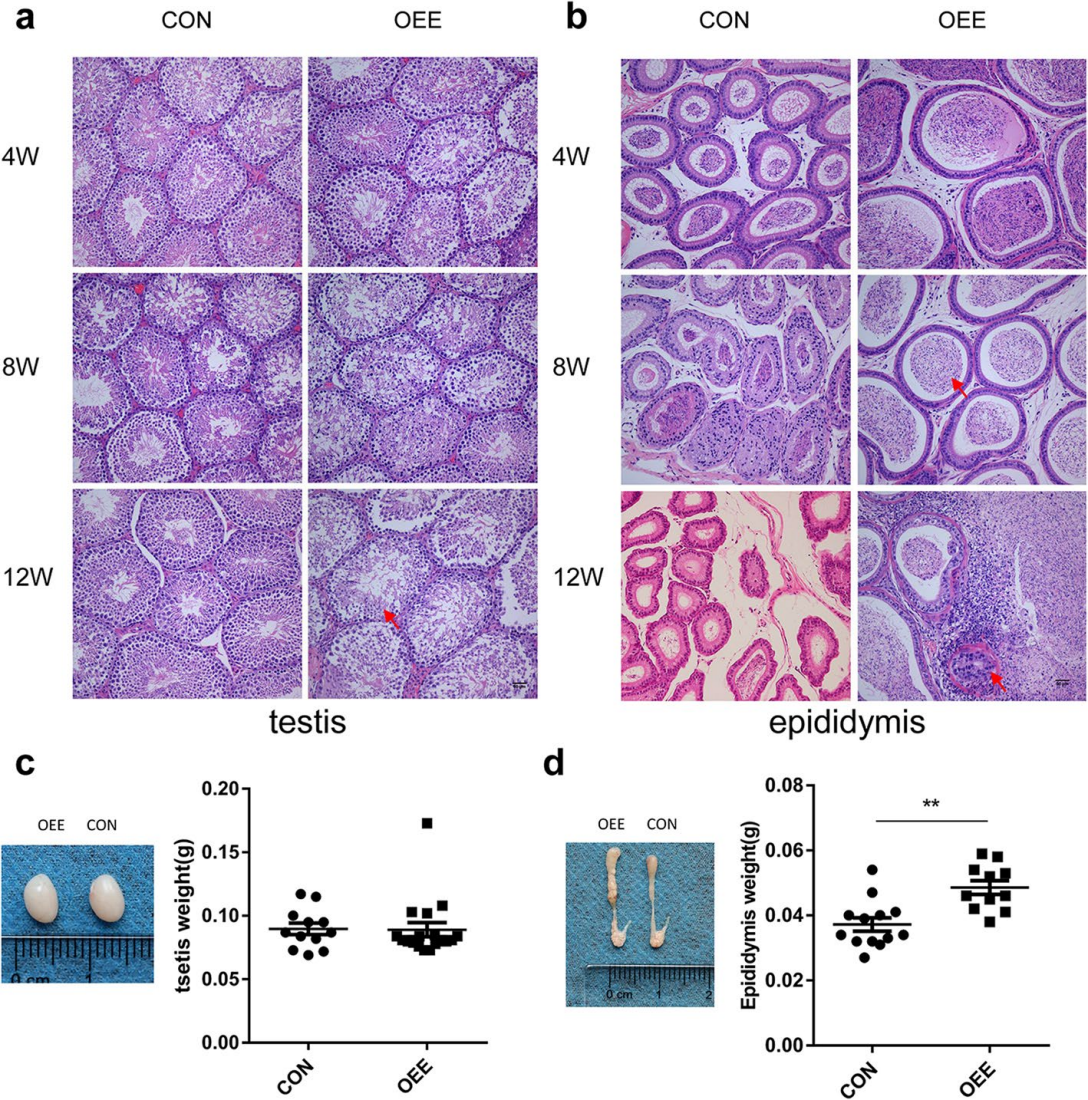
The establishment of the OEE mice model. **a** Histological testis sections from postoperative 4-,8-and12-week CON and OEE group mice. The arrow refers to the atrophied seminiferous epithelium. (× 200, scale bar = 50 μm, *N* = 3 animals/group). **b** Histological epididymis sections from postoperative 4-,8-and 12-week CON and OEE group mice. The arrow refers to atypical epididymis tubules and floccules. (× 200, scale bar = 50 μm, *N* = 3 animals/group). **c** Testis weight of postoperative 8-week CON and OEE group mice (*N* = 12 animals/CON, *N* = 18 animals/OEE). **d** Epididymis weight of postoperative 8-week CON and OEE group mice (*N* = 13 animals/CON, *N* = 11 animals/OEE).** *p*-value < 0.01

Firstly, we observed the morphology of testis and epididymis of postoperative 4-, 8-, 12- week mice to determine the proper obstructive interval via HE staining. In postoperative 4-week mice, the morphology of testes and epididymis in the OEE group showed no differences compared to the CON group. In postoperative 8-week mice, only the epididymis became markedly swollen in the OEE group. However, in 12-week postoperative mice of the OEE group, plenty of atypical epididymis tubules appeared in the epididymis, with a cluster of floccules residing in the cavity. Moreover, the number of seminiferous tubules decreased considerably, and the seminiferous epithelium atrophied in the testis (Fig. 1a,b). According to the case–control trial and the OA definition (OA is the absence of sperm in the ejaculate due to occlusion of the male reproductive tract but with the normal endocrine function) [1, 29], we chose the postoperative 8-week OEE group mice for the subsequent study. Afterward, we weighed their epididymis and testis. Compared to the CON group, the OEE group showed a significant increase in the epididymis weight, while no difference was found in the testis weight (*P* < 0.05, Fig. 1c,d).

### The obstructive epididymal environment results in poor sperm quality

**Fig. 2:**
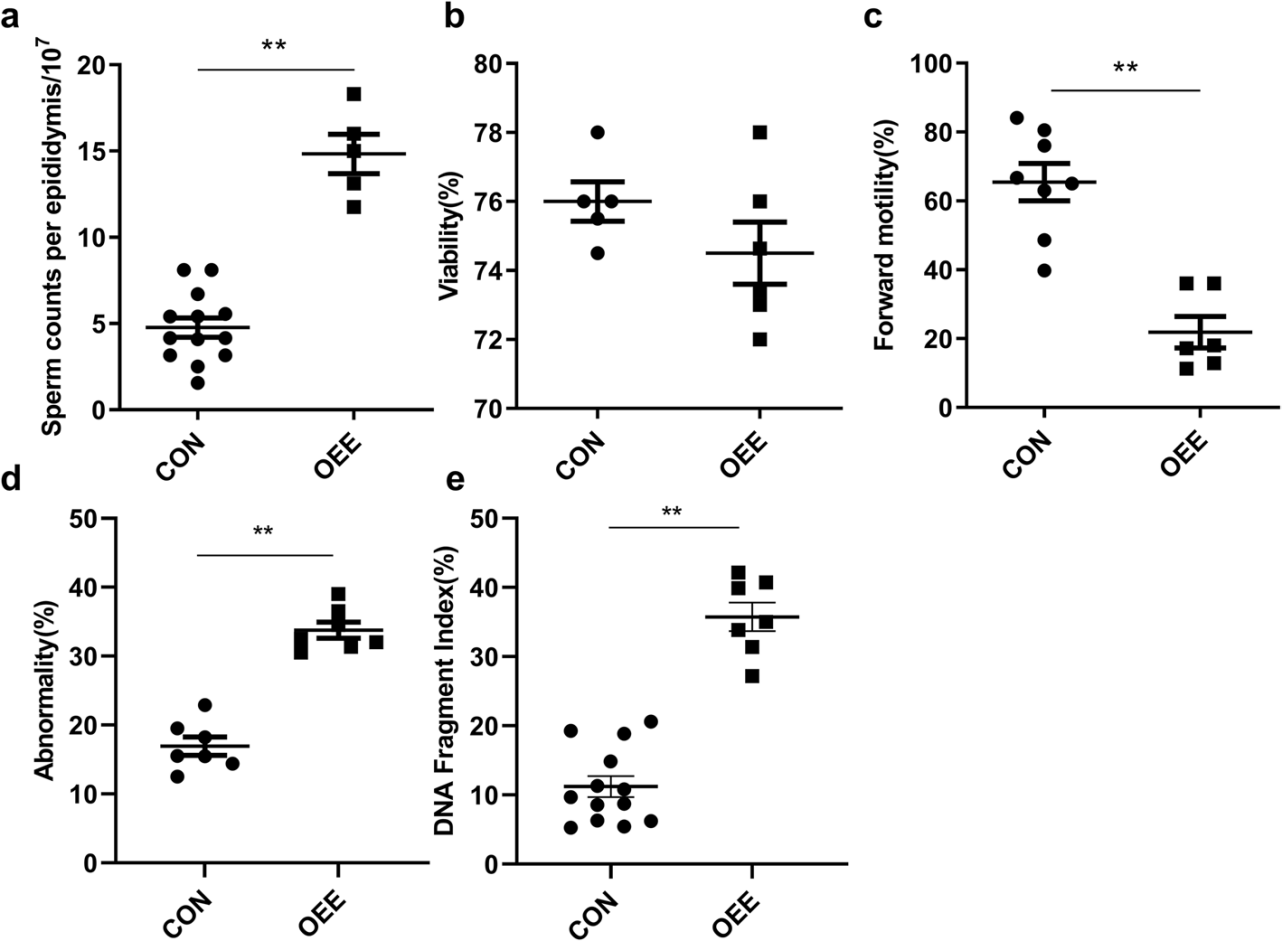
Comparison of sperm quality between the CON and OEE group mice. **a** Sperm concentration comparison (*N* = 13 animals/CON, *N* = 5 animals/OEE). **b** Sperm viability comparison (*N* = 5 animals/CON, *N* = 6 animals/OEE). **c** Sperm forward motility comparison (*N* = 8 animals/CON, *N* = 6 animals/OEE). **d** Sperm abnormality comparison (*N* = 7 animals/group). **e** Sperm DFI comparison (*N* = 13 animals/CON, *N* = 7 animals/OEE). **P-value < 0.01

The sperm concentration in the OEE group increased significantly (*P* < 0.01, Fig. 2a) compared to the CON group, as did the count of aberrant sperm (*P* < 0.01, Fig. 2d). DNA fragment index (DFI) was also significantly higher than the CON group (*P* < 0.01, Fig. 2e). The viability and forward motility of sperm decreased (*P* < 0.01, Fig. 2b,c).

### The obstructive epididymal environment results in abnormal preimplantation embryonic development which can be saved by CON group epididymosomes

**Fig. 3:**
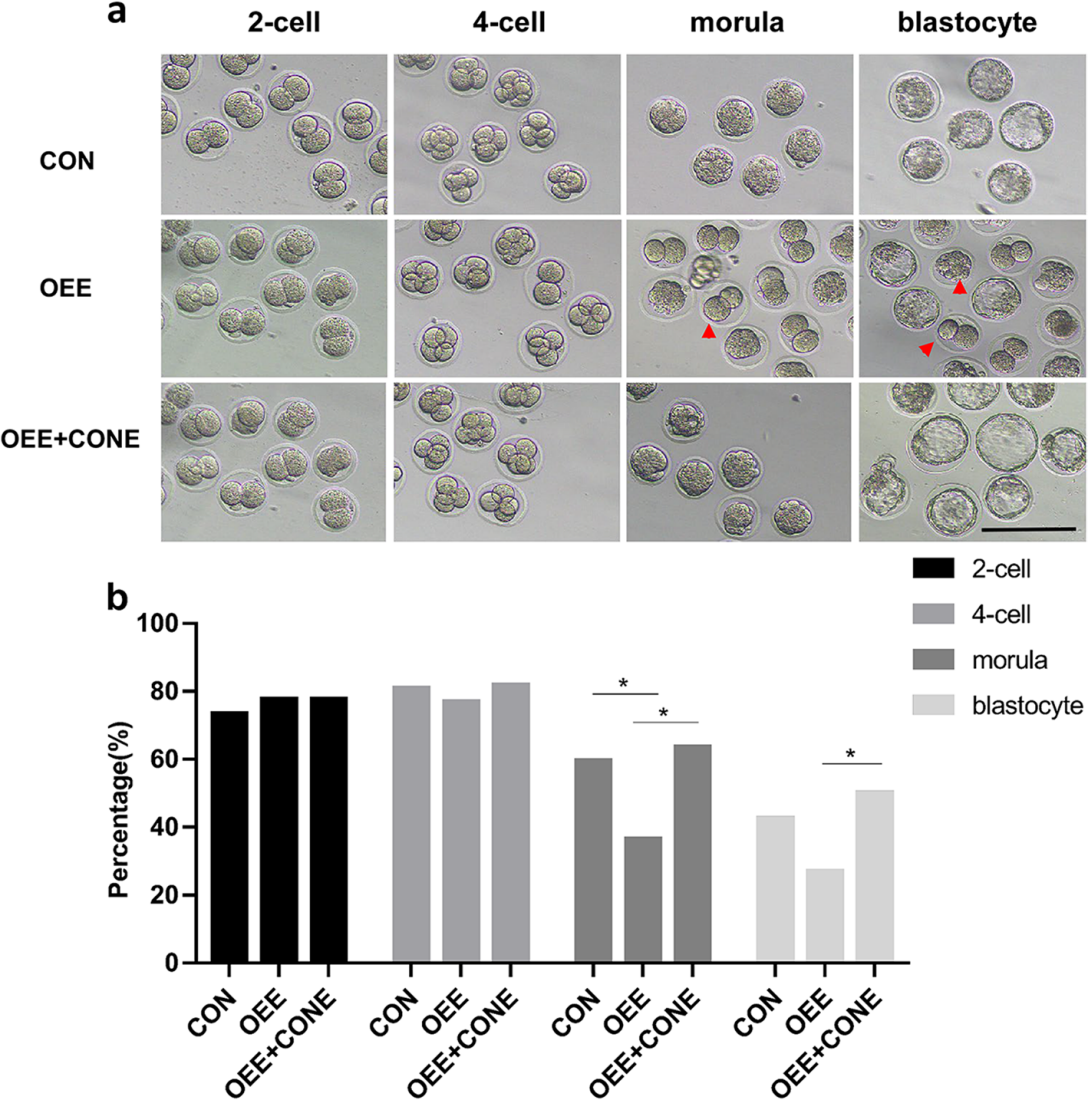
Preimplantation embryonic development of the CON, OEE, and OEE + CONE group mice. **a** Representative images of preimplantation embryonic development of CON, OEE, and OEE + CONE group mice, arrow refers to developmental arrest (× 200, scale bar = 100 μm). **b** The histogram shows the preimplantation embryonic development rate of the CON, OEE, and OEE + CONE group mice (*N* > 60 embryos/group). CONE refers to CON group epididymosomes,*p-value < 0.05

Due to the poor quality of sperm, we performed ICSI to observe preimplantation embryonic development. Our results showed that the rate of morula in the OEE group decreased compared to the CON group (*P* < 0.05), as was the rate of the blastocyst, with no statistical significance (Fig. 3). The current study examined whether an abnormal epididymal environment caused the aberrant preimplantation embryonic development; sperm from the OEE group was retrieved and incubated with epididymosomes from the CON group for subsequent ICSI (OEE + CONE group). Compared to the OEE group, the rate of the morula and blastocyst from the OEE + CONE group showed a considerable increase (*P* < 0.01 or *P* < 0.05, Fig. 3), indicating that epididymosomes from the CON group could, at least partially, save the aberrant embryonic development.

### The obstructive epididymal environment results in abnormal post-preimplantation embryonic development

**Fig. 4:**
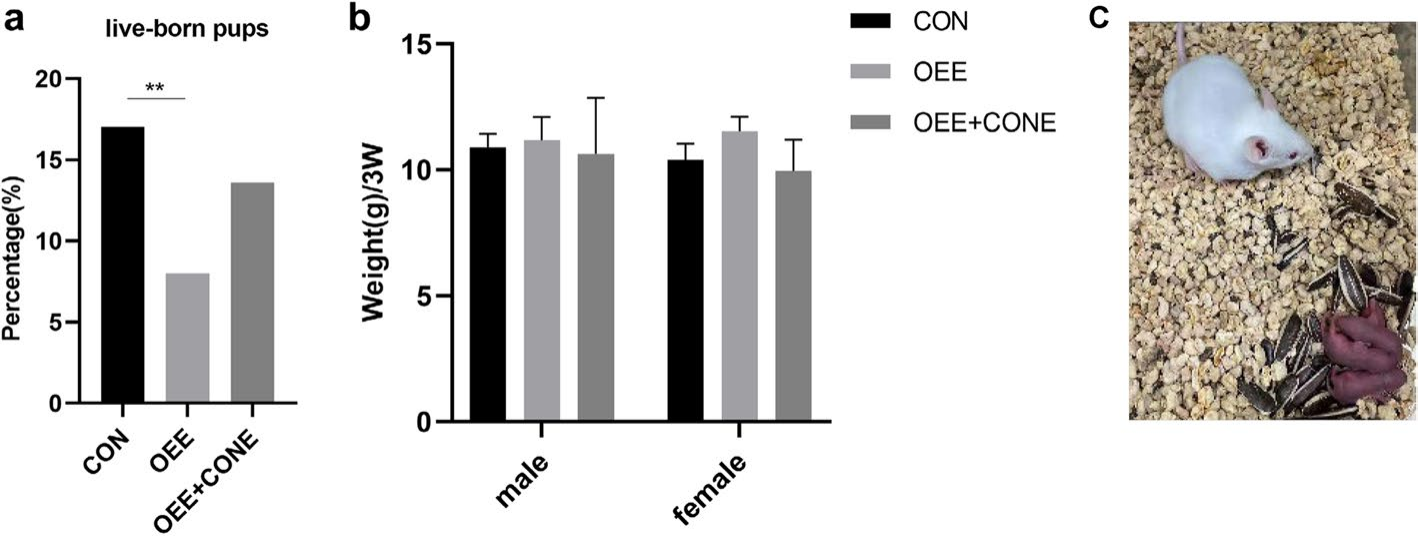
Post-preimplantation embryonic development of the CON, OEE, and OEE + CONE group mice. **a** Live-born pups of the mice of the CON, OEE, and OEE + CONE group (*N* > 180 embryos/group; *N* = 31 animals/CON, *N* = 16 animals/OEE and *N* = 26 animals/OEE + CONE). **b** The weight of 3-week-old female or male offspring from the CON, OEE, and OEE + CONE group mice (*N* = 13 male and 9 female animals /CON, *N* = 7 male and 8 female animals/OEE, *N* = 3 male and 4 female animals/OEE + CONE). **c** Representative images of live-born pups. **p-value < 0.01

Investigating the impacts of the obstructive epididymal environment on post-implantation embryonic development, 2-cell embryos of the OEE group, CON group, and OEE + CONE group were transplanted into the surrogate female ICR mice. The rate of live-born pups from the OEE group was significantly lower in comparison to the CON group (*P* < 0.05). Though there was a slight increase in the number of live-born pups from the OEE + CONE group compared to the OEE group, the difference was not statistically significant (Fig. 4a). And no difference in weight was observed between the three groups of mice, including female and male offspring, at three weeks of age (Fig. 4b,c).

### The obstructive epididymal environment affects sperm miRNA profile and downregulates the developmental miRNAs

**Fig. 5:**
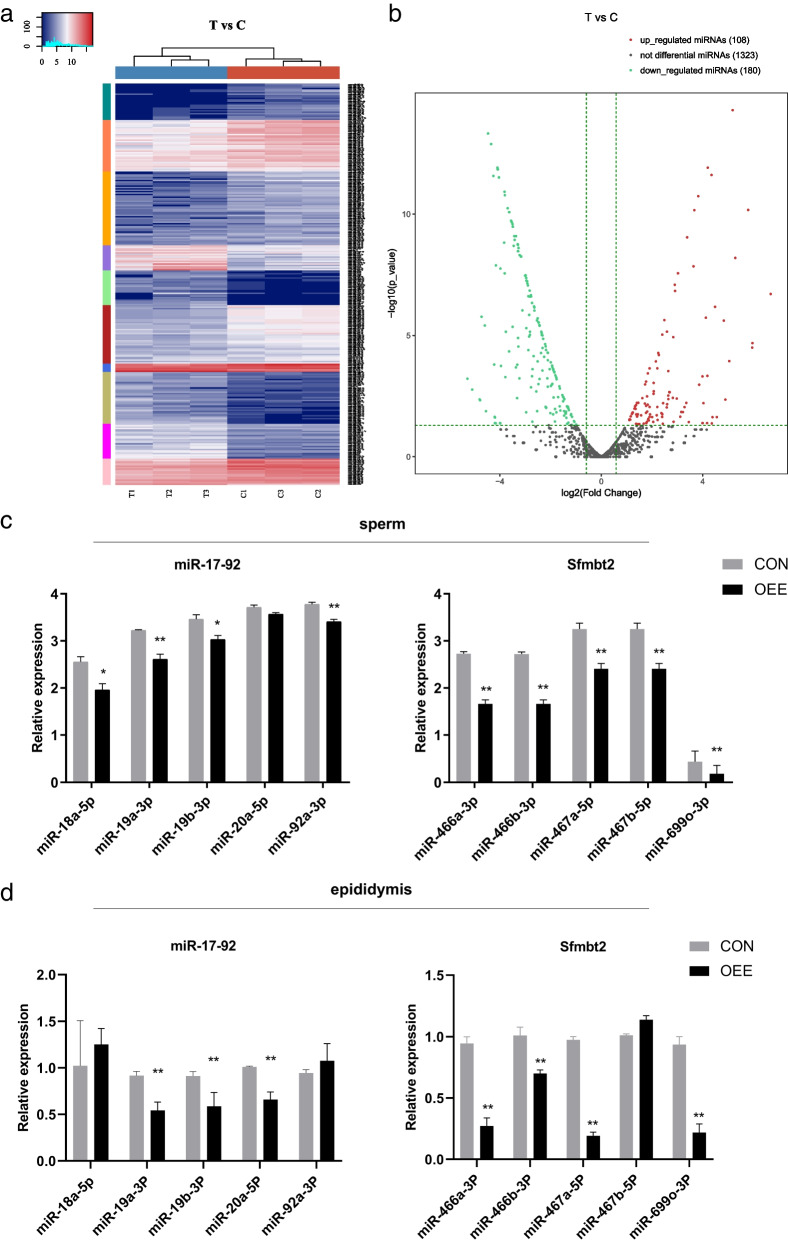
The alteration of sperm miRNA profile. **a** Heat map of sperm miRNAs differentially expressed between the CON and OEE group mice. **b** Volcano plot of sperm miRNAs differentially expressed between the CON and OEE group mice. **c** The expression of the miR-17–92 cluster and Sfmbt2 miRNA cluster in sperm from the CON and OEE group mice (*N* = 9 animals/group). **d** The expression of miR-17–92 cluster and Sfmbt2 miRNA cluster in epididymis from the CON and OEE group mice (*N* = 8 animals/group). T refers to the OEE group, C refers to the CON group, miR-17–92 and Sfmbt2 respectively refer to miR-17–92 cluster and Sfmbt2 miRNA cluster. *p-value < 0.05 or **p-value < 0.01

To examine whether sperm miRNA profile is altered under the obstructive epididymal environment, we examined sperm sRNA profile by sRNA sequencing (Supplementary Table 4). The sRNA sequencing showed that among the 1611 miRNAs detected, 594 were up-regulated, and 605 were down-regulated in the OEE group (Fig. 5a-b, Supplementary Table 5, Supplementary Fig. 1). The miR-17–92 cluster and Sfmbt2 miRNA cluster were previously reported to be related to embryonic development. Loss of the miR-17–92 cluster in mice led to lung hypoplasia and ventricular septal defect, and newborns died shortly after birth [30]. A bunch of miRNAs mainly expressed in the placenta (Supplementary Fig. 2) is located in the intron 10 of *Sfmbt2,* and knockout of the intron 10 of *Sfmbt2* resulted in placental malformation and fetal death [31, 32]. Therefore, we examined the expression of the miR-17–92 cluster and Sfmbt2 miRNA cluster in sperm and epididymis (log_2_fold change < -1 and *P* < 0.05). Compared to the CON group, the expression of the miR-17–92 cluster and Sfmbt2 miRNA cluster decreased significantly in sperm of the OEE group (*P* < 0.05 or *P* < 0.01, Fig. 5c), as well as epididymis (*P* < 0.05 or *P* < 0.001, Fig. 5d). These results demonstrated that the obstructive epididymal environment influenced the sperm miRNA profile.

### The obstructive epididymal environment regulates the expression of miRNAs in the epididymis

**Fig. 6:**
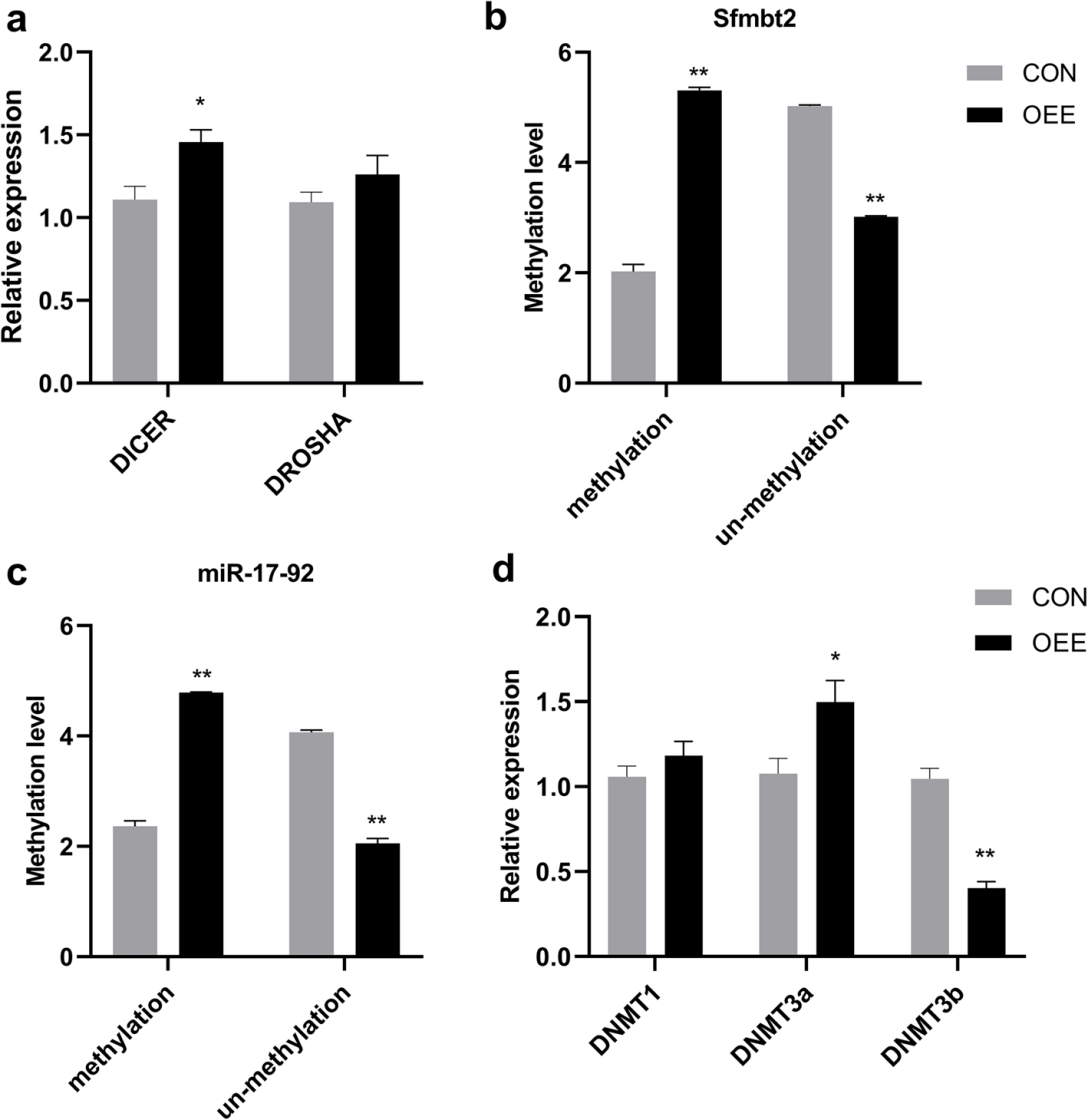
The regulation of miRNAs expression in the epididymis. **a** The expression of DROSHA and DICER mRNA in epididymis from the CON and OEE group mice (*N* = 8 animals/groups). **b**-**c** The methylation level of *MIR17HG* and *Sfmbt2* promoter-associated CpG-rich regions between the CON and OEE group (*N* = 11 animals/group). **d** The expression of DNMT1, DNMT3a, and DNMT3b mRNA in the epididymis from the CON and OEE group mice (*N* = 8 animals/groups). *p-value < 0.05 or **p-value < 0.01

To explore whether the epididymis regulated the biogenesis of miRNAs, we examined the expression of DICER and DROSHA mRNA in the epididymis. The expression of DICER and DROSHA mRNA increased in the OEE group. However, only the DICER enzyme had a significant difference (*P* < 0.05, Fig. 6a). Since the methylation of promoter-associated CpG-rich regions also regulates the expression of miRNAs, we also measured the methylation level of promoter-associated CpG-rich regions of the two miRNA clusters. And the methylation level of promoter-associated CpG-rich regions increased in the OEE group compared to the CON group (*P* < 0.05 or *P* < 0.01, Fig. 6b,c). We determined the expression of the maintenance of DNA methylation enzymes DNMT1, de novo DNA methylation enzymes DNMT3a and DNMT3b mRNA in the epididymis. The expression of DNMT3a mRNA in the OEE group increased significantly and DNMT3b mRNA decreased significantly (*P* < 0.05 or *P* < 0.01, Fig. 6 d). The abundance of the miR-17–92 cluster and Sfmbt2 miRNA cluster was regulated by DNA methylation.

### The miR-17–92 cluster and Sfmbt2 miRNA cluster inhibitors do not impair preimplantation embryonic development

**Fig. 7:**
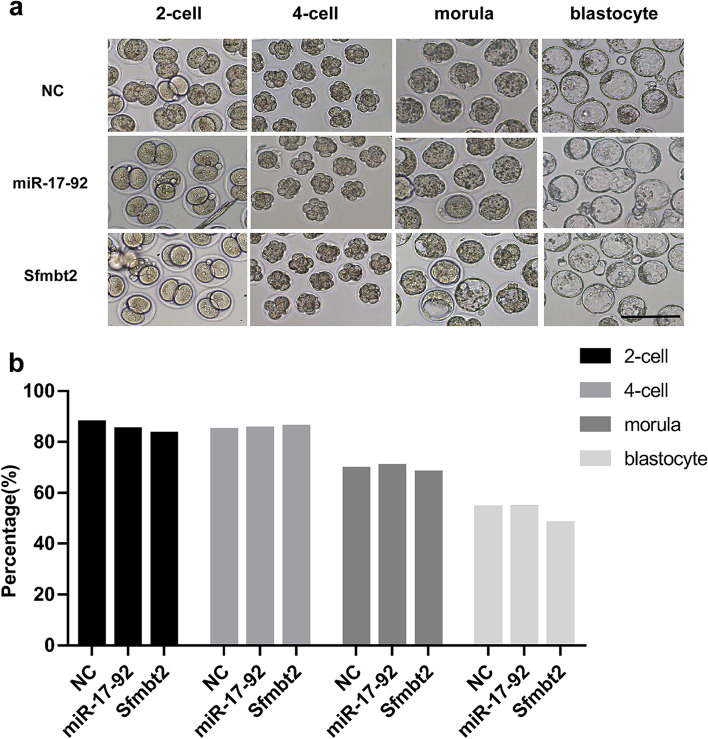
The preimplantation embryonic development of zygotes microinjected with miR-17–92 cluster or Sfmbt2 miRNA cluster inhibitors. **a** Representative images of preimplantation embryonic development of the NC, miR-17–92, and Sfmbt2 group (× 200, scale bar = 100 μm). **b** Histogram showing the rate of preimplantation embryonic development of the NC, miR-17–92, and Sfmbt2 group (*N* > 60 embryos/group). NC refers to the negative control, miR-17–92 and Sfmbt2 respectively refers to miR-17–92 cluster inhibitors group and Sfmbt2 miRNA cluster inhibitors group

To verify whether the downregulation of the expression of the miR-17–92 cluster and Sfmbt2 miRNA cluster would result in aberrant embryonic development, we injected the inhibitors of the miR-17–92 cluster and Sfmbt2 miRNA cluster into normal zygotes. The results showed that compared to the negative group, the downregulation of the miR-17–92 cluster and Sfmbt2 miRNA cluster didn’t impair embryonic development (Fig. 7, Supplementary Fig. 3).

### The miR-17–92 cluster and Sfmbt2 miRNA cluster inhibitors do not impair post-implantation embryonic development

**Fig.8:**
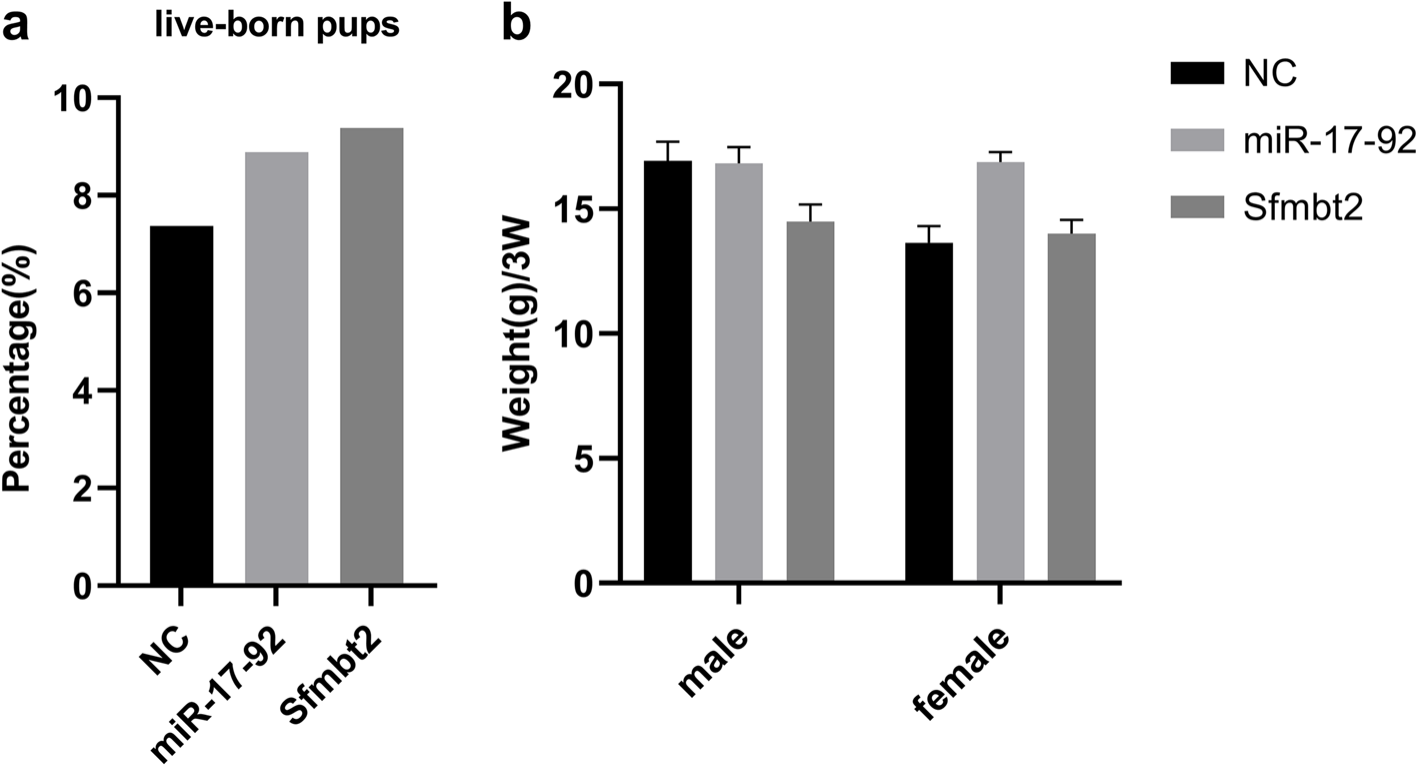
The post-implantation embryonic development of zygotes microinjected with miR-17–92 cluster or Sfmbt2 miRNA cluster inhibitors. **a** Live-born pups of the NC, miR-17–92, and Sfmbt2 group (*N* > 90 embryos/group; *N* = 7 animals/NC, *N* = 8 animals/miR-17–92 and *N* = 9 animals/Sfmbt2). **b** The weight of 3-week-old female or male offspring from the NC, miR-17–92, and Sfmbt2 group (*N* = 3 male and 4 female animals/NC, *N* = 3 male and 3 female animals/miR-17–92 and *N* = 3 female and 5 male animals/Sfmbt2). NC refers to the negative control, miR-17–92 and Sfmbt2 respectively refer to miR-17–92 cluster inhibitors group and Sfmbt2 miRNA cluster inhibitors group

We transferred the 2-cell embryos of the NC group, miR-17–92 group, and Sfmbt2 group into the female ICR surrogate mice. There was no difference in the rate of live-born pups and the weight of 3-week-old female and male offspring compared to the negative group (Fig. 8). Together, these results indicated that the downregulation of the miR-17–92 cluster or Sfmbt2 miRNA cluster alone might not be sufficient to impair post-implantation embryonic development.

### The combination of miR-17–92 cluster and Sfmbt2 miRNA cluster inhibitors microinjection does not affect post-implantation embryonic development

**Fig. 9:**
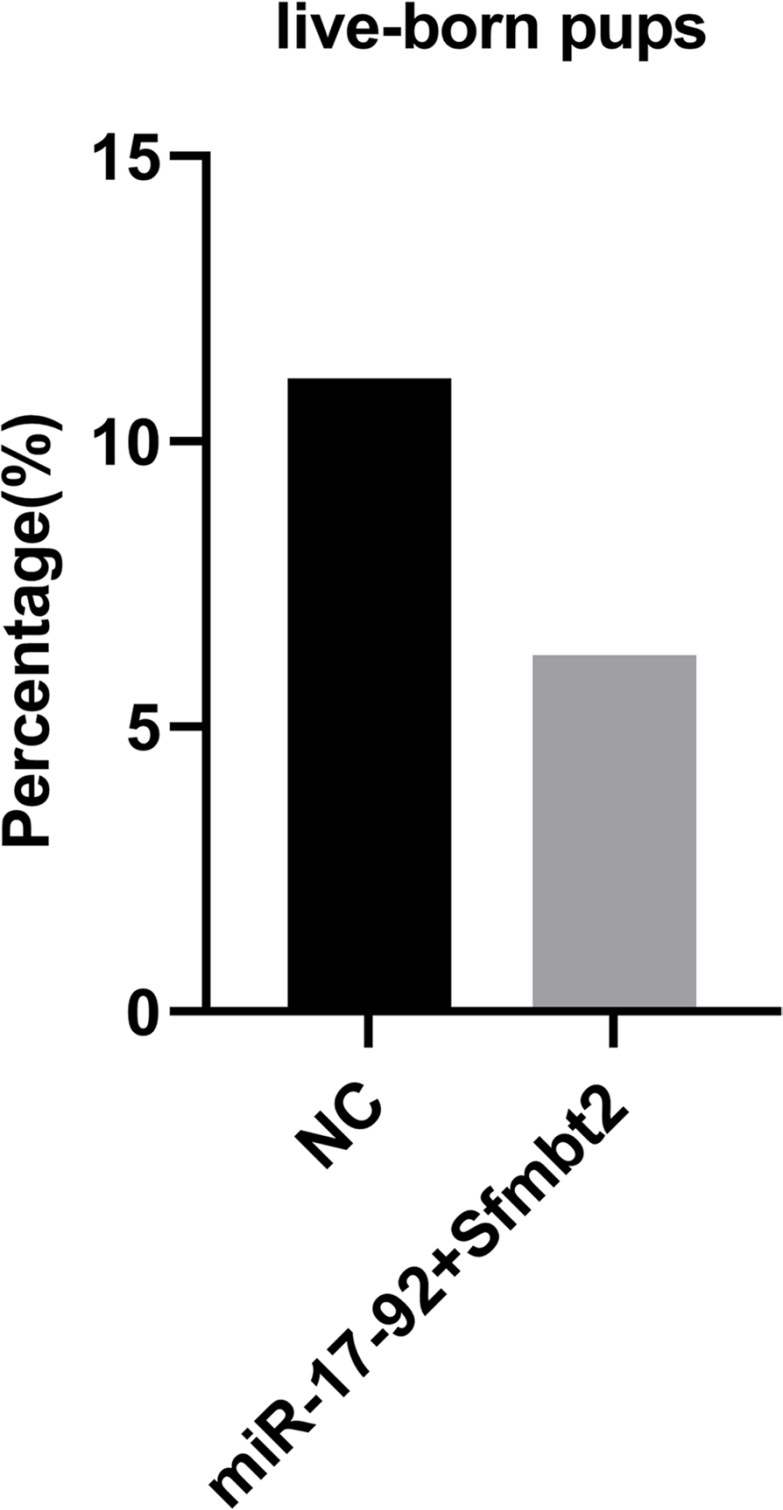
The post-implantation embryonic development of zygote microinjected with miR-17–92 cluster and Sfmbt2 miRNA cluster (*N* > 90 embryos/group; *N* = 10 animals/NC, *N* = 6 animals/miR-17–92 + Sfmbt2). NC refers to the negative control, miR-17–92 + Sfmbt2 refers to miR-17–92 cluster and Sfmbt2 miRNA cluster inhibitors group

Given that single miRNA cluster is not sufficient to impair embryonic development, we mixed the inhibitors of miR-17–92 cluster and the Sfmbt2 miRNA cluster to inject into normal zygotes and transferred the 2-cell embryo to ICR surrogate mice. There was no difference in the rate of live-born pups compared to the negative group, indicating that the miR-17–92 cluster and Sfmbt2 miRNA cluster were not the cause of abnormal embryonic development (Fig. 9).

## Discussion

Our results showed that the obstructive epididymal environment affected sperm quality and resultant embryonic development. Furthermore, The obstructive epididymal environment can regulate the abundance of the developmental miR-17–92 cluster and the Sfmbt2 miRNA cluster in sperm by DNA methylation. However, the downregulation of their expression in normal zygotes did not hinder embryonic development.

Studies of obstructive epididymis mainly focused on the reproductive outcomes of OA patients. Our study developed the mice model to mimic the human’s obstructive epididymal environment for the first time and explored the mechanism of the obstructive epididymal environment on sperm quality and resultant embryonic development. In population data, the obstructive epididymal environment is found to harm sperm quality. The first post-vasectomy sperm analysis for postoperative vasectomy patients revealed that the sum of motile sperm decreased sharply [33]. Quality tests for OA patients’ epididymal sperm showed DFI, and aberrant sperm increased [34]. Our findings confirmed these results; additionally, unvital sperm increased in OEE group mice. Some studies reported that embryo quality of OA epididymal sperm degraded because of obstruction. Ali Buran’s and Shih’s studies showed that the epididymal sperm had poor embryo quality compared to testicular sperm in OA patients [9, 35]. And our study also showed the developmental potential of the OEE group decreased. Nonetheless, Solomon’s population study showed no difference in the embryonic development of epididymal sperm between the OA group and the donor sperm group [10]. The obstructive interval was reported to be positively correlated with pregnancy rate and not mentioned in their study [36]. The obstructive interval might be a reason for the discrepancy between our study and Solomon’s. Through the epdidymosomes fusion experiment, We further confirmed the effect of obstructive epididymis on embryonic development in mice model.

Mature sperm was transcriptionally and translationally silent due to lack of ribosomes and highly compacted DNA; sperm RNA originated from remnants of spermatogenesis and epididymal transit [37]. The miRNA profiles of sperm, tissues and epdidymosomes from different epididymis segments reveals that miRNAs transmitted from epididymis to sperm are essential to embryonic development and offspring’s long-term health [16, 38]. Moreover, the abnormal epididymal environment changed the abundance of developmental miRNAs in sperm. Alshanbayeva et al. revealed early stress life influenced the abundance of sperm miR-34c, which is reported to be related to the first cleavage of embryos [39]. Chan et al. reported that miRNAs transmitted from epididymis to sperm in a chronic stress mice model could duplicated the paternal stress phenotype in offspring [40]. The NGS for the epididymis from vasectomized and normal patients and seminal microvesicles from normal, vasectomized, vasovasostomized patients revealed the miRNAs profiles had a significant change in epididymis and epididymosomes (seminal microvesicles mainly consist in epididymosomes) [41]. Our study also revealed the change of developmental miR-17–92 cluster and Sfmbt2 miRNA cluster in sperm under an obstructive epididymal environment.

DROSHA and DICER process the biogenesis of RNAs and the knockout of DROSHA and DICER decreases the biogenesis of most miRNAs [42]. Li’s study showed the expression of the miR-17–92 cluster was positively correlated with the expression of DICER and DROSHA [43]. This is not consistent with our findings that the abundance of miR-17–92 cluster and Sfmbt2 miRNA cluster were downregulated but DICER and DROSHA were upregulated. DNA methylation is an epigenetic mark involved in the repression of transcription. The methylation level of CpG-island at transcriptional start sites is correlated with transcription activity [44]. DNMT1, DNMT3a, and DNMT3b are canonical cytosine-5 DNA methyltransferases that catalyze the addition of methylation marks to genomic DNA [45]. Duaa’s study showed the hypermethylation of the miR-17–92 cluster promoter resulted in a significant downregulation of the miR-17–92 cluster in idiopathic pulmonary fibrosis patients, and the methylation level was upregulated by the DNMT1 [46]. The upregulation of DNMT3a repressed the expression of miRNAs [47]. Our study revealed the hypermethylation of the promoter of the miR-17–92 cluster and the upregulation of the expression of DNMT3a and DNMT1 in the epididymis. Considering the transcriptional inertia of mature sperm, we speculated that epididymis regulated the expression of miR-17–92 cluster and Sfmbt2 miRNA cluster.

Collins’s study suggested caput sperm led to abnormal post-implantation embryonic development due to a lack of some key miRNAs transmitted from epididymis [13, 38, 48] (miR-880 cluster, the paralogous miR-17–92, miR-106b-25 clusters, and the miR-34b/c pair), and suppletion of these miRNAs to the embryo of caput sperm rescued the abnormal post-implantation embryonic development [12, 49]. Our study found developmental miR-17–92 cluster and Sfmbt2 miRNA clusters decreased. MiR-17–92 cluster is made up of MiR-17, miR-18a, miR-19a, miR-20a, miR-19b-1, and miR-92a-1 and is involved in the cell cycle, proliferation, apoptosis, and other processes [50]. Humans with heterozygous microdeletions in the *MIR17HG* locus had severe skeletal abnormalities [51]. The deletion of* MIR17HG* in mice resulted in perinatal lethality, and germline deletion of the miR-17–92 cluster recapitulated the phenotype observed in humans, such as growth restriction, cardiac defects, and lung hypoplasia [52]. Furthermore, the miR-17–92 cluster was discovered to be required for B cell development and lymphocyte homeostasis [30]. The *Sfmbt2* gene is paternally expressed in the mouse placenta and is required for placental development; *Sfmbt2* knockout mice failed to maintain trophoblasts [53]. The specifical deletion of the entire Sfmbt2 miRNA cluster in mice resulted in placental malformation, especially in the spongiotrophoblast layer [31]; furthermore, deletion of H3K27me3 imprinting in the Sfmbt2 miRNA cluster enlarged mouse placentas [54]. In our study, the downregulation of the developmental miR-17–92 cluster and Sfmbt2 miRNA cluster in normal zygotes did not hinder embryonic development. Unlike Collins’s study, in which a bunch of different miRNAs were injected into zygotes and influenced embryonic development, we only injected two clusters of miRNAs. And it seems that these miRNA clusters microinjection into zygotes are insufficient to affect embryonic development. In addition, differences in embryos derived from the caput sperm and cauda sperm have been questioned, which might be resulted from the methods used in the preparation of the sperm head rather than the miRNAs [55, 56]. Besides, we can’t rule out whether proteins transmitted from epididymosomes have an impact on embryonic development. The studies of epididymosomes proteins reveals that epididymosomes proteins are related to sperm function, such as motility, capacitation, acrosome reaction, and so on [57]. And the sperm cell contains a large number of proteins involved in regulating translation and transcription that might also be critical for early embryos [58]. Above all, the function of the epididymis and sperm miRNAs in early embryonic development is still not very clear and warrants further exploration.

## Conclusion

In conclusion, our findings show that an obstructive epididymal environment influences sperm quality and resultant embryonic development, as well as sperm miRNA profile, indicating that the epididymis is vital in early embryonic development and may play a potential role in sperm epigenome. Although the downregulation of the miR-17–92 cluster and Sfmbt2 miRNA clusters in zygotes did not hinder embryonic development, our findings provide evidence to support further research into the effects of sperm miRNAs on embryonic development.

## Supplementary Information

**Additional file 1: Supplementary Table 5.** The list of miRNAs expression in Sequencing data.

**Additional file 2: Supplementary Table 1.** Primers sequences for RT-qPCR. **Supplementary Table 2.** Primers sequences for DNA methylation. **Supplementary Table 3.** RNA sequences for microinjection. **Supplementary Table 4.** Sequencing quality score.

**Additional file 3: Supplementary figure 1. **The bioinformatics analysis of sperm sRNA sequencing **a** Heatmap of correlation coefficient of sperm sRNAs from the CON and OEE group. **b** Principal component (PC) analysis of sperm sRNAs from the CON and OEE group. **c** Comparison of the proportion of sperm miRNAs and tsRNAs from the CON and OEE group. **d** The GO enrichment analysis of differentially expressed miRNAs that were down-regulated or up-regulated in the OEE group. **e** KEGG pathway analysis of differentially expressed miRNAs that were down-regulated or up-regulated in the OEE group. C refers to CON group, T refers to OEE group. **Supplementary figure 2. **The overview of the Sfmbt2 miRNA cluster profile (Sfmbt2 miRNA cluster profile can be browsed using NCBI database, https://www.ncbi.nlm.nih.gov/gene). **Supplementary figure 3.** The validation of the function of the anti-sense oligos **a**. The expression of miR-92a-3p in HIN3T3 cells transfected with miR-92a-3p inhibitor or negative control inhibitor. (*N*=3 samples/group). **b**. The expression of target genes mRNA of miR-92a-3p in HIN3T3 cells transfected with miR-92a-3p inhibitor or negative control inhibitor. (*N*=3 samples/group). The relative expression of genes was normalized to the level of Gapdh, *p-value <0.05, NC refers to the negative control inhibitor and inhibitor refers to miR-92a-3p inhibitor.

## Data Availability

The Sequencing data supporting this study's findings have been deposited in National Genomics Data Center (NGDC) with accession codes CRA007699.

## Abbreviations

miRNAs: MicroRNAs
OEE: Obstructive epididymal environment
CON: Control
HE: Hematoxylin and eosin
ICSI: Intracytoplasmic sperm injection
OA: Obstructive azoospermia
NGS: The high throughput sequencing
sRNAs: Small RNAs
pri-miRNAs: Primary miRNAs
pre-miRNAs: Precursor miRNAs
cKO: Conditional knockout
DFI: DNA fragmentation index
PMSG: Pregnant mare serum gonadotropin
hCG: Human chorionic gonadotrophin
COCs: Cumulus-oocyte complexes
RT-qPCR: Real-time quantitative polymerase chain reaction
snRNA: Small nuclear RNA
Actb: Beta-actin
