# Supplementary material for: The developmental miR-17–92 cluster and the Sfmbt2 miRNA cluster cannot rescue the abnormal embryonic development generated using obstructive epididymal environment-producing sperm in C57BL/6 J mice
Source: Reprod Biol Endocrinol. 2022 Nov 30;20:164. doi: 10.1186/s12958-022-01025-x (PMC9710060; doi:10.1186/s12958-022-01025-x)
Supplement: Supplementary file 2 — Additional file 2: Supplementary Table 1. Primers sequences for RT-qPCR. Supplementary Table 2. Primers sequences for DNA methylation. Supplementary Table 3. RNA sequences for microinjection. Supplementary Table 4. Sequencing quality score. [file 12958_2022_1025_MOESM2_ESM.docx]

**Supplementary Table 1. Primers sequences for RT-qPCR**

| MiRNA/Gene | Primers sequences |
| --- | --- |

| miR-18a-5P | UAAGGUGCAUCUAGUGCAGAUAG |
| --- | --- |
| miR-19a-3P | UGUGCAAAUCUAUGCAAAACUGA |
| miR-19b-3P | UGUGCAAAUCCAUGCAAAACUGA |
| miR-20a-5P | UAAAGUGCUUAUAGUGCAGGUAG |
| miR-92a-3P | UAUUGCACUUGUCCCGGCCUGU |
| miR-466a-3P | TATACATACACGCACACATAAGA |
| miR-466b-3P | ATACATACACGCACACATAAGA |
| miR-467a-5P | TAAGTGCCTGCATGTATATGCG |
| miR-467b-5P | GTAAGTGCCTGCATGTATATG |
| miR-669o-3P | ACATAACATACACACACACGTAT |
| DNMT1 forward | AGAGACCAGGATAAGAAACGCA |
| DNMT1 reverse | CTCCTTTGATTTCCGCCTCAAT |
| DNMT3a forward | GATGAGCCTGAGTATGAGGATGG |
| DNMT3a reverse | CAAGACACAATTCGGCCTGG |
| DNMT3b forward | CGTTAATGGGAACTTCAGTGACC |
| DNMT3b reverse | CTGCGTGTAATTCAGAAGGCT |
| Dicer forward | GGTCCTTTCTTTGGACTGCCA |
| Dicer reverse | GCGATGAACGTCTTCCCTGA |
| Drosha forward | ATGCAAGGCAATACGTGTCAT |
| Drosha reverse | TTTTGGGGTCTGAAAGCTGGT |
| Mapk8 F | GTGGAATCAAGCACCTTCACT |
| Mapk8 R | TCCTCGCCAGTCCAAAATCAA |
| Cpeb4 F | GCCCCGGCTACTCATAACATT |
| Cpeb4 R | TCAGGCAGTATTCCAGCTTCC |
| Gapdh F | GGAGAGTGTTTCCTCGTCCC |
| Gapdh R | ACTGTGCCGTTGAATTTGCC |
| Actb forward | GGCTGTATTCCCCTCCATCG |
| Actb reverse | CCAGTTGGTAACAATGCCATGT |
|  |  |

**Supplementary Table 2. Primers sequences for DNA methylation**

| MiRNA | Primers sequences |  |  |
| --- | --- | --- | --- |
| miRNA17-92-M sense | GTCGATGTAGAGTTTGCGTGGTG |  |  |
| miRNA17-92-M Antisense | ACTAAACGATAACCAAACGAAACTAAA |  |  |
| miRNA17-92-UM sense | UGUGCAAAUCUAUGCAAAACUGA |  |  |
| miRNA17-92-UM Antisense | UGUGCAAAUCCAUGCAAAACUGA |  |  |
| Sfmbt2-M sense | TTTCGGGAGTGGTAAATTTCGG |  |  |
| Sfmbt2-M Antisense | CTTTAACTCTAAAACGTCTCGCCTT |  |  |
| Sfmbt2-UM sense | TTTTGGGAGTGGTAAATTTTGGT |  |  |
| Sfmbt2-UM Antisense | AATTCTTTAACTCTAAAACATCTCACCTT |  |  |

**Supplementary Table 3. RNA sequences for microinjection**

| MiRNA | RNA sequences |
| --- | --- |
| NC inhibitor | CAGUACUUUUGUGUAGUACAA |
| mmu-miR-18a-5p inhibitor | CUAUCUGCACUAGAUGCACCUUA |
| mmu-miR-19a-3p inhibitor | UCAGUUUUGCAUAGAUUUGCACA |
| mmu-miR-92a-3p inhibitor | CAGGCCGGGACAAGUGCAAUA |
| mmu-miR-20a-5p inhibitor | CUACCUGCACUAUAAGCACUUUA |
| mmu-miR-467a-5p inhibitor | CGCAUAUACAUGCAGGCACUUA |
| mmu-miR-467b-5p inhibitor | CAUAUACAUGCAGGCACUUAC |
| mmu-miR-466a-3p inhibitor | UCUUAUGUGUGCGUGUAUGUAUA |
| mmu-miR-466b-3p inhibitor | UCUUAUGUGUGCGUGUAUGUAU |
| mmu-miR-669o-3p inhibitor | AUACGUGUGUGUGUAUGUUAUGU |

**Supplementary Table 4. Sequencing quality score**

| Sample | Total Read | Total Base | BaseQ30 | BaseQ30(%) |
| --- | --- | --- | --- | --- |
| C1 | 10890116 | 555395916 | 519220268 | 93.49 |
| C2 | 12419469 | 633392919 | 591637449 | 93.41 |
| C3 | 10674172 | 544382772 | 508645893 | 93.44 |
| T1 | 9845304 | 502110504 | 467509546 | 93.11 |
| T2 | 11527264 | 587890464 | 548004636 | 93.22 |
| T3 | 11026362 | 562344462 | 522899224 | 92.99 |

**Sample: C refers to the CON group and T refers to the OEE group**

**Total reads: raw sequencing reads after quality filtering**

**Total Base: number of bases after quality filtering**

**Base Q30: number of bases of Q score more than 30 after quality filtering**

**Base Q30(%): the proportion of bases (Q 30) number after quality filtering**
