# Supplementary material for: The developmental miR-17–92 cluster and the Sfmbt2 miRNA cluster cannot rescue the abnormal embryonic development generated using obstructive epididymal environment-producing sperm in C57BL/6 J mice
Source: Reprod Biol Endocrinol. 2022 Nov 30;20:164. doi: 10.1186/s12958-022-01025-x (PMC9710060; doi:10.1186/s12958-022-01025-x)
Supplement: Supplementary file 3 — Additional file 3: Supplementary figure 1. The bioinformatics analysis of sperm sRNA sequencing a Heatmap of correlation coefficient of sperm sRNAs from the CON and OEE group. b Principal component (PC) analysis of sperm sRNAs from the CON and OEE group. c Comparison of the proportion of sperm miRNAs and tsRNAs from the CON and OEE group. d The GO enrichment analysis of differentially expressed miRNAs that were down-regulated or up-regulated in the OEE group. e KEGG pathway analysis of differentially expressed miRNAs that were down-regulated or up-regulated in the OEE group. C refers to CON group, T refers to OEE group. Supplementary figure 2. The overview of the Sfmbt2 miRNA cluster profile (Sfmbt2 miRNA cluster profile can be browsed using NCBI database, https://www.ncbi.nlm.nih.gov/gene). Supplementary figure 3. The validation of the function of the anti-sense oligos a. The expression of miR-92a-3p in HIN3T3 cells transfected with miR-92a-3p inhibitor or negative control inhibitor. (N=3 samples/group). b. The expression of target genes mRNA of miR-92a-3p in HIN3T3 cells transfected with miR-92a-3p inhibitor or negative control inhibitor. (N=3 samples/group). The relative expression of genes was normalized to the level of Gapdh, *p-value <0.05, NC refers to the negative control inhibitor and inhibitor refers to miR-92a-3p inhibitor. [file 12958_2022_1025_MOESM3_ESM.pdf]

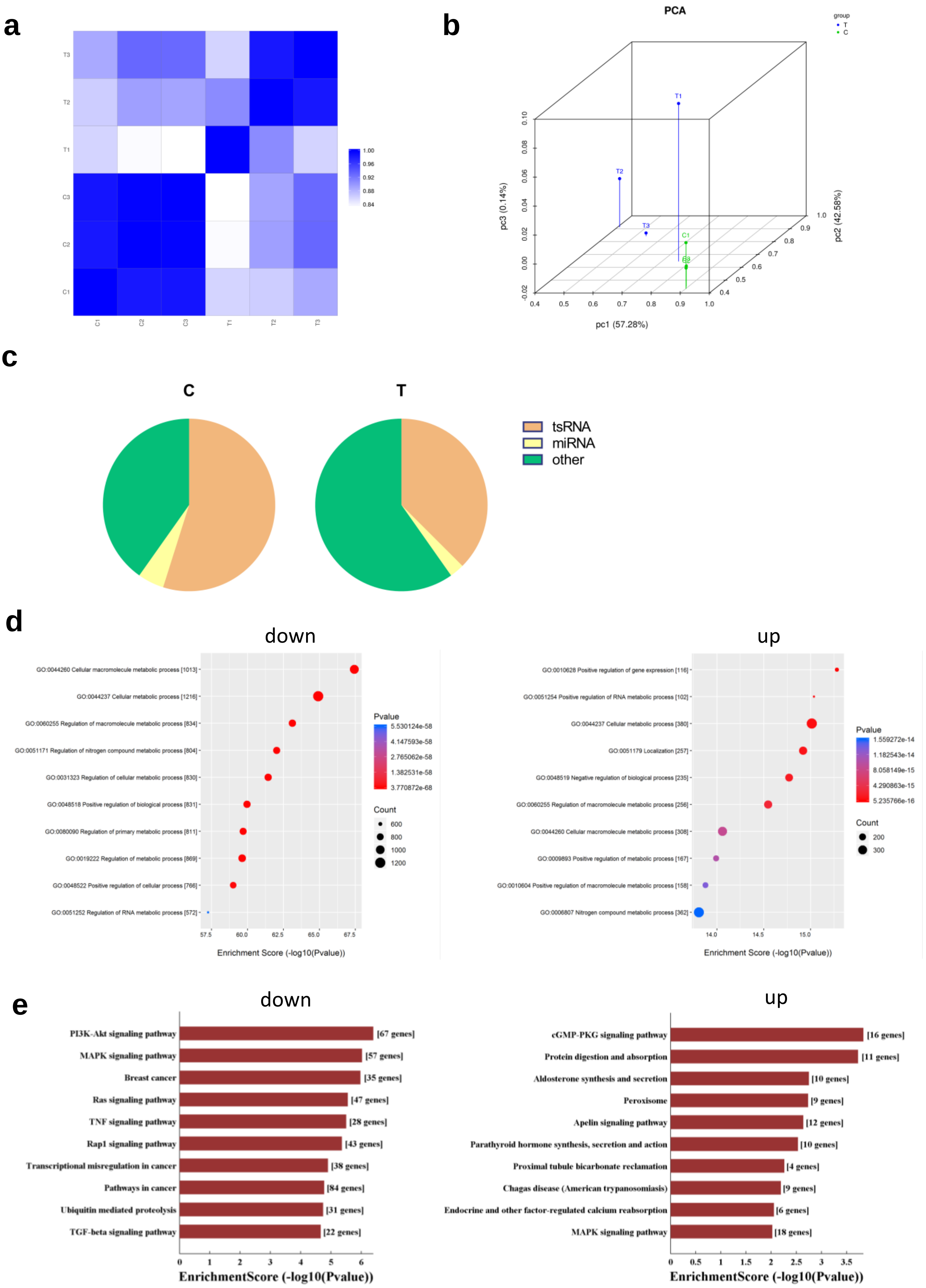

Supplementary figure 1. The bioinformatics analysis of sperm sRNA sequencing. **a** Heatmap of correlation coefficient of sperm sRNAs from the CON and OEE group. **b** Principal component (PC) analysis of sperm sRNAs from the CON and OEE group.**c** Comparison of the proportion of sperm miRNAs and tsRNAs from the CON and OEE group. **d** The GO enrichment analysis of differentially expressed miRNAs that were down-regulated or up-regulated in the OEE group.**e** KEGG pathway analysis of differentially expressed miRNAs that were down-regulated or up-regulated in the OEE group. C refers to CON group, T refers to OEE group.

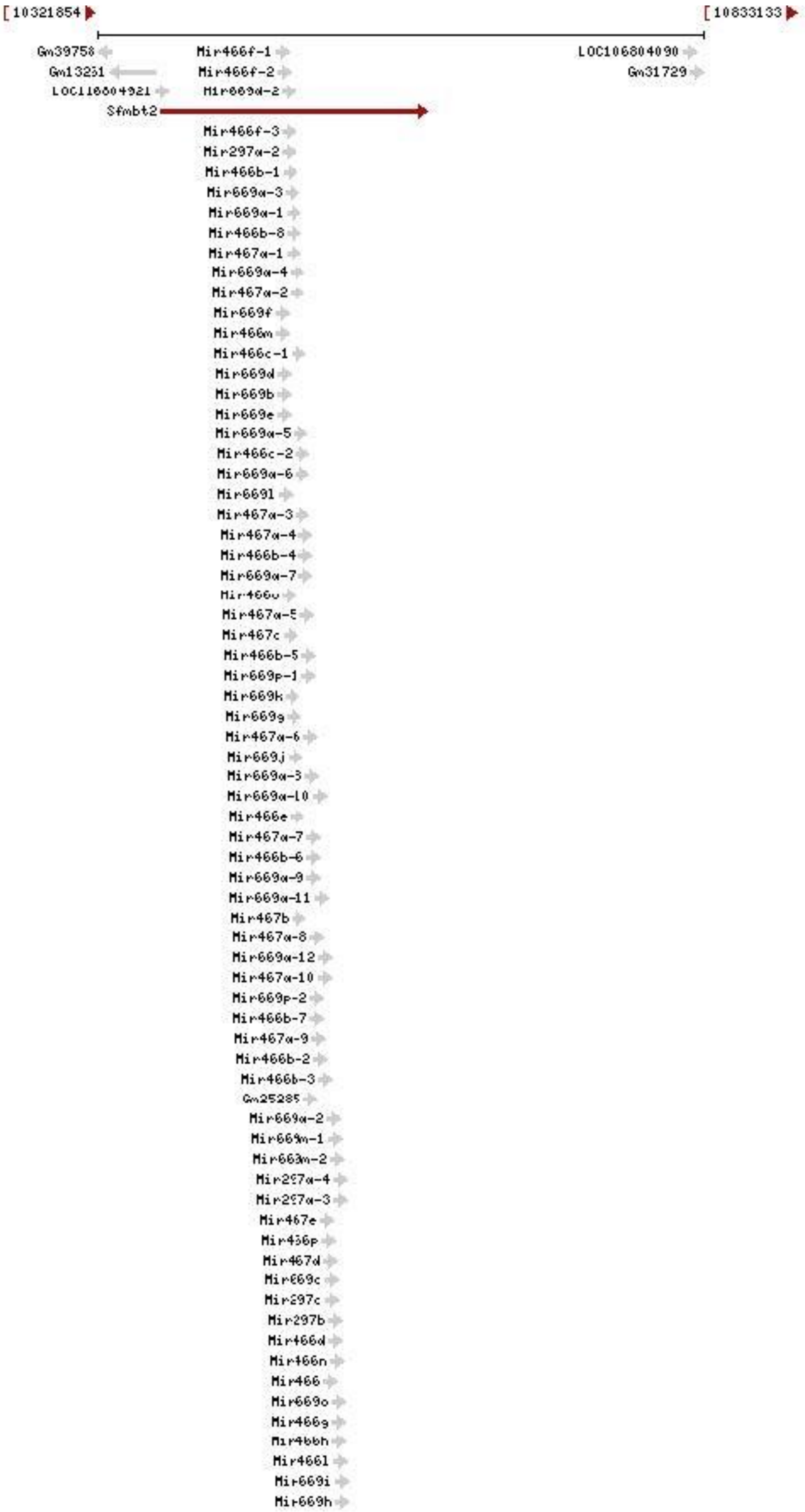

Supplementary figure 2.The overview of the Sfmbt2 miRNA cluster profile (Sfmbt2 miRNA cluster profile can be browsed using NCBI database, https://www.ncbi.nlm.nih.gov/gene).

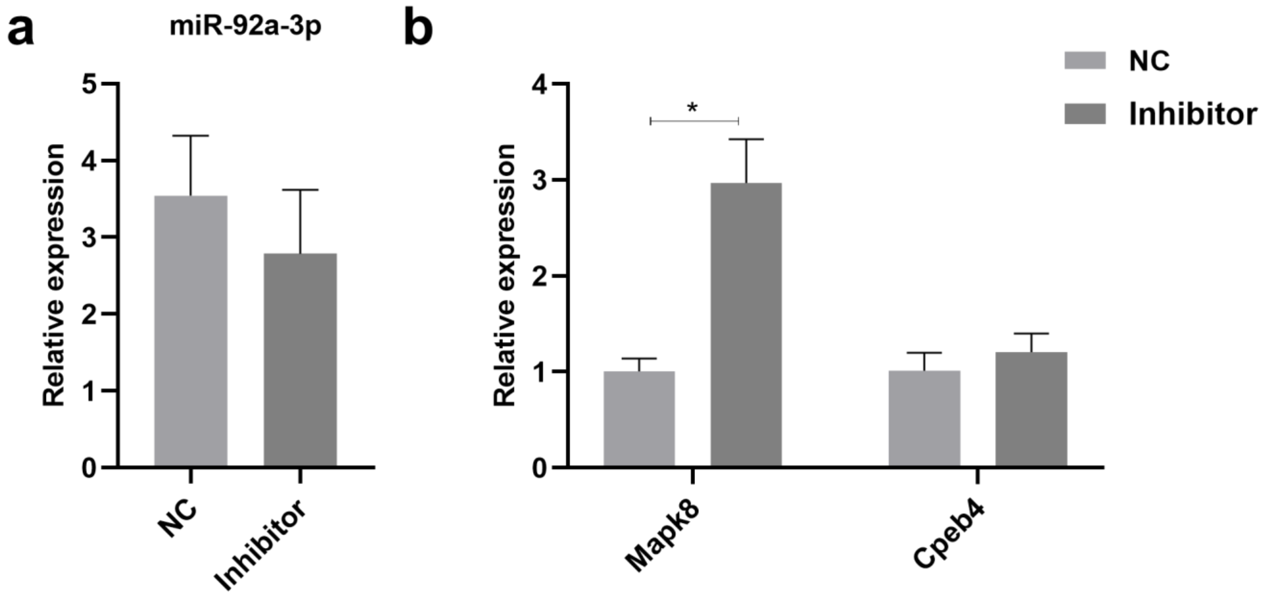

Supplementary figure 3. The validation of the function of the anti-sense oligos **a**. The expression of miR-92a-3p in HIN3T3 cells transfected with miR-92a-3p inhibitor or negative control inhibitor. (N=3 samples/group). **b**. The expression of target genes mRNA of miR-92a-3p in HIN3T3 cells transfected with miR-92a-3p inhibitor or negative control inhibitor. (N=3 samples/group). The relative expression of genes was normalized to the level of Gapdh, **p*-value <0.05, NC refers to the negative control inhibitor and inhibitor refers to miR-92a-3p inhibitor
